# Disrupted Sensorimotor Network Integration in Women With Fibromyalgia Revealed by Resting‐State Functional MRI

**DOI:** 10.1111/jon.70118

**Published:** 2026-01-10

**Authors:** Gina Rodrigues de Oliveira, Tamires Morett Gama, Lucas Rego Ramos, Marcos Fabio DosSantos

**Affiliations:** ^1^ Postgraduate Program in Anatomical Pathology Federal University of Rio de Janeiro (UFRJ) Rio de Janeiro Brazil; ^2^ Laboratory of Cell Morphogenesis, Biomedical Sciences Institute Federal University of Rio de Janeiro (UFRJ) Rio de Janeiro Brazil; ^3^ Postgraduate Program in Radiology Federal University of Rio de Janeiro (UFRJ) Rio de Janeiro Brazil; ^4^ Postgraduate Program in Translational Neuroscience Federal University of Rio de Janeiro (UFRJ) Rio de Janeiro Brazil

**Keywords:** chronic pain, fibromyalgia, functional connectivity, resting‐state fMRI, sensorimotor network

## Abstract

**Background and Purpose:**

Fibromyalgia (FM) is a chronic syndrome characterized by widespread musculoskeletal pain, hypersensitivity, and cognitive impairments. Alterations in brain functional connectivity have been suggested as possible mechanisms underlying pain amplification in these patients. This study aimed to investigate patterns of brain functional connectivity in patients with FM using resting‐state functional magnetic resonance imaging.

**Methods:**

Data were obtained from the public OpenNeuro repository and acquired on a 3 Tesla scanner. The sample consisted of 33 women with a clinical diagnosis of FM (*x̅* = 41.73 ± 6.09 years) and 33 age‐matched healthy controls (*x̅* = 41.52 ± 6.03 years), with no significant differences in age (*p* = 0.89) or education level (*p* = 0.81). Images were processed and analyzed using independent component analysis. Between‐group comparisons were corrected for multiple comparisons using false discovery rate (FDR) correction (*p* < 0.05).

**Results:**

Patients with FM showed a significant reduction in functional connectivity within the right sensorimotor network (SMN) compared to controls (*p*‐FDR < 0.05). Moreover, a negative correlation was observed between connectivity in this network and the sensory dimension of pain assessed by the McGill Pain Questionnaire (*r* = −0.35; *p* = 0.05).

**Conclusion:**

The reduced functional connectivity within the SMN may represent a neurobiological marker of FM, reflecting dysfunctions in sensorimotor integration and central modulation of pain. These findings support the hypothesis that FM involves functional brain alterations related to pain perception and amplification.

## 1 Introduction

Fibromyalgia (FM) is a highly prevalent chronic pain syndrome characterized by persistent widespread pain and additional symptoms such as fatigue, sleep disturbances, and cognitive impairment [1]. Its prevalence ranges from 0.2% to 6.6% in the general population and from 2.4% to 6.8% among women [2]. The condition is most common between 35 and 60 years of age, and its occurrence is up to eight times higher in women than in men [2]. In clinical settings, prevalence may reach 15% [3]. FM carries a substantial socioeconomic burden, leading to disability in daily activities, limited workforce participation, reduced social engagement, increased healthcare costs, and loss of productivity [1]. This considerable psychosocial burden makes detection and management particularly challenging.

The etiology of FM is multifactorial and remains controversial. No organic cause or specific biomarker has been identified; thus, diagnosis relies exclusively on clinical criteria and subjective patient reports. The American College of Rheumatology revised its diagnostic framework in 2010, eliminating “tender points” as a diagnostic requirement [4] and replacing them with broader measures of pain and associated symptoms [5]. Multiple genetic, environmental, neuroendocrine, and psychosocial factors are believed to contribute to its development [6, 7], and up to 50% of susceptibility may be heritable, though no single physiological mechanism fully accounts for the disorder [6, 8].

Neurophysiological models emphasize abnormalities in the central nervous system, particularly central sensitization, a pathological state in which minimal nociceptive input produces exaggerated pain responses [9]. As a result, patients experience allodynia and hyperalgesia even in the absence of tissue damage [10]. Evidence also points to dysfunction in pain modulation systems, including increased excitatory neurotransmitters (glutamate, substance P) and decreased inhibitory neurotransmitters (serotonin, norepinephrine, endogenous opioids), leading to amplified pain processing [11, 12]. In addition, altered inhibitory circuitry reduces the CNS's ability to suppress nociceptive signals [11, 12, 13]. Structural and functional abnormalities have been described in pain‐related regions such as the insula, anterior cingulate cortex, and prefrontal cortex [11, 13, 14]. Together, these findings support the notion that FM represents a brain‐based disorder, driven predominantly by central mechanisms of pain amplification [11, 15].

Functional connectivity analysis enables investigation of spontaneous temporal correlations in neural activity at rest or during tasks, allowing identification of functional networks and characterization of network‐level abnormalities. Several studies have demonstrated altered resting‐state connectivity in FM, including disrupted communication between the default mode network (DMN) and regions involved in pain, cognition, and emotion [16]. These findings reveal widespread alterations in the functional architecture of the FM brain and help clarify how chronic pain becomes consolidated within the CNS.

The sensorimotor network (SMN) integrates discriminative aspects of pain—such as location and quality—with motor responses and muscle tone. Prior evidence indicates decreased connectivity between pain‐related areas and sensorimotor regions in FM [17]. On the basis of this literature, we hypothesize that alterations in motor and somatosensory cortices may contribute to clinical pain in FM. Therefore, our study aims to specifically investigate SMN functional connectivity in patients with FM and determine whether disruptions in this network are related to pain intensity.

## 2 Methods

### 2.1 Participants

Data for this study were obtained from the openly accessible dataset ds004144 (version 1.0.0) available on the OpenNeuro platform (https://openneuro.org/datasets/ds004144/versions/1.0.0), released under a CC0 license. The original study was approved by the Ethics Committee of the Instituto Nacional de Psiquiatría “Ramón de la Fuente Muñiz” (Mexico City), and all participants provided written informed consent.

The sample consisted exclusively of women, including patients diagnosed with FM and healthy female controls matched for sociodemographic characteristics. Groups were individually matched for age (FM: 41.73 ± 6.09 years; controls: 41.52 ± 6.03 years; *p* = 0.89) and education level (*p* = 0.81), ensuring comparable demographic profiles. All participants were between 30 and 55 years old and reported no history of neurological conditions, psychiatric disorders, head trauma, or MRI contraindications. FM diagnoses followed the 2016 Revised American College of Rheumatology criteria. Control participants were recruited from the same population and screened to confirm absence of chronic pain or neurological symptoms.

### 2.2 McGill Pain Questionnaire (MPQ)

Clinical pain assessment was conducted using the MPQ, a widely recognized instrument for measuring the multidimensional experience of pain. Originally developed by Ronald Melzack in the 1970s, the MPQ is considered one of the most comprehensive tools for evaluating both chronic and acute pain [18], as it integrates sensory, affective, and evaluative components of the pain experience.

The questionnaire consists of a standardized list of verbal descriptors organized into categories representing different facets of pain. Participants are instructed to select, within each category, the terms that best describe their pain experience. Each descriptor is associated with a predetermined numerical value, allowing the calculation of dimension‐specific scores.

The MPQ assesses three principal dimensions of pain: (i) sensory dimension—reflects the physical and perceptual characteristics of pain, including location, intensity, temporal pattern, and sensory quality (e.g., throbbing, burning, and tingling); (ii) affective dimension—captures the emotional responses associated with pain, such as distress, exhaustion, or suffering (e.g., tiring, punishing, and unbearable); (iii) evaluative dimension—represents the individual's overall subjective judgment of pain intensity (e.g., mild, moderate, and severe).

In addition to these core dimensions, the MPQ includes a miscellaneous category composed of supplementary descriptors that contribute to the overall characterization of pain. Each selected descriptor is assigned a numerical score, reflecting the relative intensity of that pain quality. Summed scores for each dimension yield the final subscale values used in the statistical analyses.

### 2.3 MRI Data and Preprocessing

Resting‐state functional MRI data were acquired on a 3.0 Tesla MRI system using a blood‐oxygen‐level‐dependent (BOLD) sensitive echo‐planar imaging sequence. The acquisition protocol consisted of a matrix of 80 × 80 × 37 voxels, yielding 300 time‐series volumes. Each voxel had an isotropic resolution of 3 × 3 × 3 mm^3^, with a repetition time (TR) of 2000 ms and an echo time (TE) of 30 ms.

Preprocessing and statistical analyses were performed using the CONN functional connectivity toolbox (version 23, Gabrieli Lab, McGovern Institute for Brain Research, Massachusetts Institute of Technology, Cambridge, MA, USA; http://www.conn‐toolbox.org) [19] implemented in SPM12 software (Functional Imaging Laboratory, UCL Queen Square Institute of Neurology, London, UK), implemented in MATLAB (MathWorks, Natick, MA, USA), and executed in MATLAB R2023b (MathWorks, Natick, MA, USA). Preprocessing followed standard resting‐state fMRI procedures, including discarding initial volumes, motion correction, slice‐timing correction, normalization to MNI space, spatial smoothing with a Gaussian kernel (full width at half maximum, FWHM = 8 mm), detrending, and nuisance regression (white matter, cerebrospinal fluid, and motion parameters). Temporal filtering was applied using a band‐pass filter of 0.008–0.09 Hz. All participants met quality criteria with framewise displacement below 0.5 mm.

Functional connectivity was examined using independent component analysis (ICA), a multivariate approach that decomposes BOLD signals into statistically independent spatial components identifying coherent intrinsic networks [20]. Between‐group comparisons were conducted at a voxel‐wise threshold of *p* < 0.001 (uncorrected) and cluster‐level threshold corrected for multiple comparisons using the false discovery rate (FDR) method, adopting *p*‐FDR < 0.05 [21].

### 2.4 Statistical Analyses

Statistical analyses were performed using the JAMOVI software (version 2.4.8.0, The jamovi project, Sydney, Australia). Comparisons of sociodemographic and psychometric data between groups were conducted using the *χ* ^2^ test for categorical variables and either the Mann–Whitney *U*‐test (for non‐Gaussian distributions) or independent‐samples Student's *t*‐test (for Gaussian distributions) for continuous variables.

## 3 Results

### 3.1 Sociodemographic and Clinical Characteristics

The FM and control groups did not differ significantly in age (*p* = 0.89) or education level (*p* = 0.81). Body mass index (BMI), however, was significantly higher in the FM group (26.9 ± 4.08 kg/m^2^) compared to controls (24.8 ± 3.16 kg/m^2^; *p* = 0.03). Median household income did not differ significantly between groups (*p* = 0.30). Disease duration in the FM group ranged from 2 to 8 years, with a median of 5 years. The sociodemographic characteristics of the sample are presented in Table 1.

**TABLE 1:** Sociodemographic and clinical characteristics.

	FM group	Control group	*p* value
Age (years)	41.73 ± 6.09	41.52 ± 6.03	0.89
Body mass index (kg/m^2^)	26.9 ± 4.08	24.8 ± 3.16	0.03^a^
Education level	*N*	*N*	
Elementary school	2	1	0.81
High school	3	1	
Technical degree	8	8	
Bachelor's degree	13	14	
Postgraduate studies	7	9	
Income (Mexican Pesos)	9.5k (4k–15k)	10k (5k–20k)	0.30
Disease duration (years)	5 (2.0–8.0)	—	—

*Note*: All the data represent mean ± standard deviation for parametric distributions and median (Q1–Q3) for nonparametric distributions, unless otherwise indicated. *N* = number of participants. Q1 = First Quartile; Q3 = Third Quartile.

Abbreviation: FM, fibromyalgia.

^a^ The statistical threshold was set at *p* < 0.05.

### 3.2 Functional Connectivity

**FIGURE 1:**
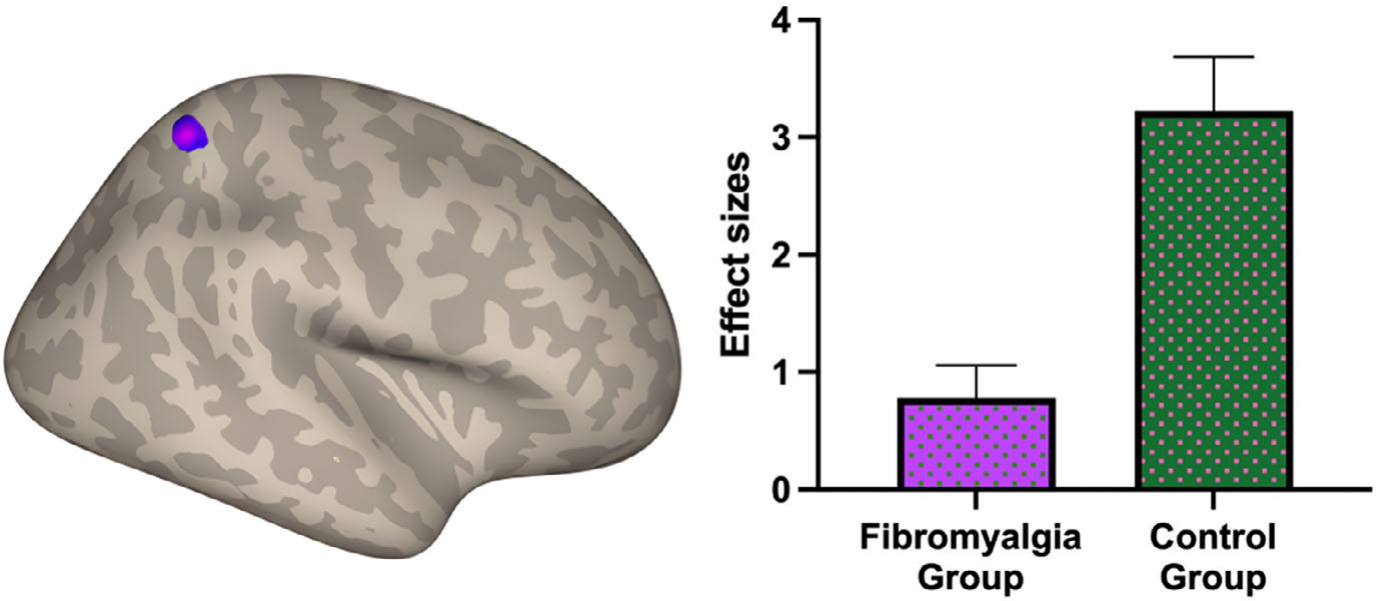
The cortical surface map (left) shows the right‐hemisphere cluster exhibiting significantly reduced functional connectivity in patients with fibromyalgia compared with healthy controls (*p*‐FDR < 0.05, voxel‐wise *p* < 0.001). The bar graph (right) displays mean functional connectivity values (±standard error) for the fibromyalgia (purple) and control (green) groups.

The functional connectivity analysis revealed a significant reduction in connectivity within the right SMN in patients with FM compared to controls. This difference was identified using ICA, applying a cluster‐level FDR correction (*p*‐FDR < 0.05) and a voxel‐wise threshold of *p* < 0.001 (Figure 1). A single significant cluster comprising 99 voxels was detected, with a peak at MNI coordinates [+36, −54, +62], located primarily in the right superior parietal lobule. The anatomical composition of the cluster included 77 voxels (78%) in the superior parietal lobule, 10 voxels (10%) in the right lateral occipital cortex (superior division), and 12 voxels (12%) in non‐labeled regions.

No significant differences were observed in the left hemisphere, nor in other intrinsic functional networks analyzed, including the default mode, executive, limbic, visual, auditory, and attentional networks.

### 3.3 Correlations Between Functional Connectivity and Pain Measures

**FIGURE 2:**
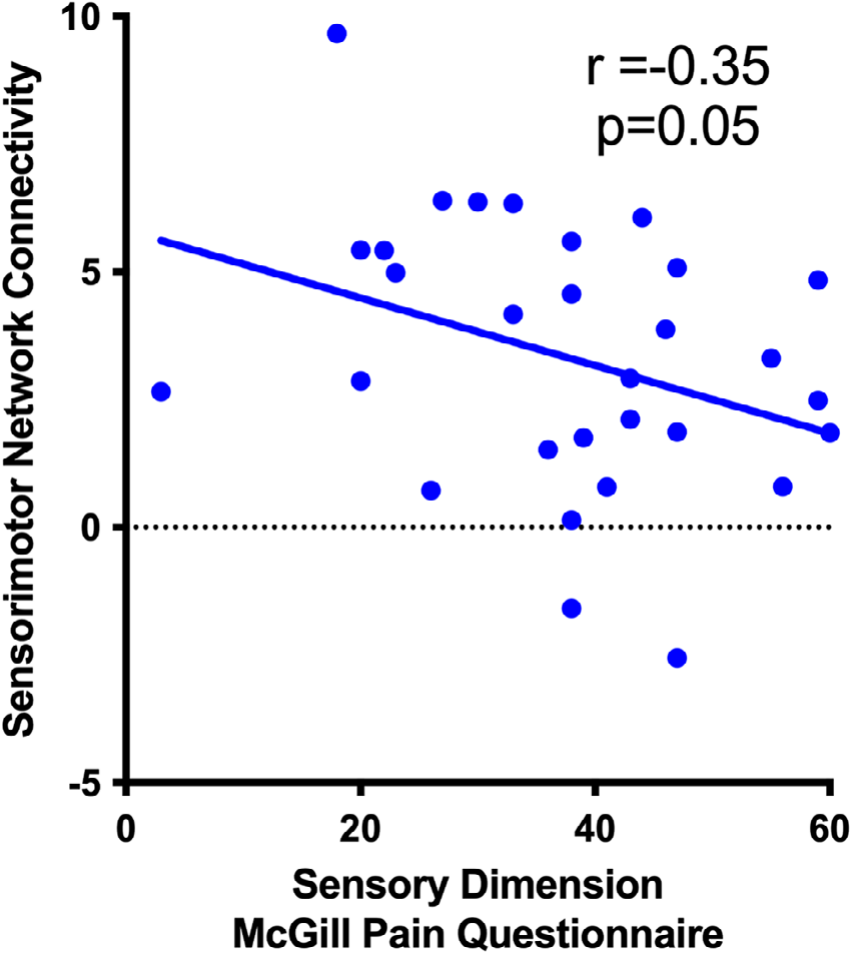
Scatter plot showing the negative correlation between functional connectivity in the right sensorimotor network and sensory pain scores from the McGill Pain Questionnaire. A significant negative association was observed (*r* = −0.35; *p* = 0.05), indicating that lower functional connectivity is related to higher sensory pain perception.

Pearson correlation analysis indicated a significant negative association between right SMN connectivity and the sensory dimension of the MPQ (*r* = −0.35; *p* = 0.05). This finding suggests that lower functional connectivity in this network is associated with higher sensory pain scores (Figure 2).

No significant correlations were found between functional connectivity and the affective (*r* = −0.32; *p* = 0.07), evaluative (*r* = −0.18; *p* = 0.31), or miscellaneous (*r* = −0.27; *p* = 0.14) McGill dimensions.

## 4 Discussion

In this study, patients with FM exhibited a significant reduction in functional connectivity within the right SMN compared to healthy controls. This decreased connectivity was negatively correlated with the sensory dimension of pain from the MPQ, suggesting that lower functional integration in this network is associated with a greater subjective perception of sensory pain. However, this association was modest in magnitude and reached only the threshold of statistical significance. No significant differences were found in other intrinsic networks, nor were relevant correlations observed for the affective, evaluative, or miscellaneous pain dimensions.

This right‐lateralized reduction in SMN connectivity mirrors the findings of our previous study using the same dataset and the same participants, where women with FM showed structural alterations specifically in the right hemisphere, including changes in the precentral gyrus, postcentral gyrus, and anterior insula [22]. The fact that both imaging modalities converge on the same neural system and the same hemisphere reinforces the robustness of the observed neural signature. This multimodal overlap suggests a pattern of sensorimotor disruption in FM, supporting the hypothesis that disturbances in sensory‐discriminative circuits contribute directly to symptom severity.

The decreased right SMN connectivity associated with higher sensory‐pain scores is consistent with the findings of Flodin [17], who reported that patients with FM exhibit reduced connectivity between pain‐processing structures and premotor and sensorimotor areas. Similarly, Pujol [23] demonstrated that self‐reported pain intensity correlates with reduced connectivity between the secondary somatosensory cortex and the primary somatosensory cortex (S1). These findings reinforce the interpretation that greater pain perception is associated with weaker functional integration at sensory levels, suggesting a disruption of neural circuits responsible for the sensory‐discriminative component of pain.

Additional evidence supports altered sensory processing in FM. Studies have shown reduced intracortical inhibition in S1, which is associated with increased clinical pain and central sensitization processes [24, 25]. Furthermore, reduced functional connectivity between S1 and regions involved in descending pain modulation, such as the periaqueductal gray (PAG), has been associated with poorer sleep quality, higher pain intensity, and stronger central sensitization symptoms [14, 24]. A potential mechanistic explanation involves increased neuroinflammation, with evidence of elevated glial activation in both motor and somatosensory cortices in patients with FM [26].

Hypoconnectivity of the SMN is not unique to FM. It is a recurring pattern across various neurological conditions and centralized pain syndromes, reflecting disruptions in sensory‐motor integration. In migraine, studies report somatotopic distortions and reduced interhemispheric SMN connectivity [27, 28]. In complex regional pain syndrome (CRPS), alterations in SMN organization have also been documented [29, 30, 31]. In chronic low back pain, large‐scale network reorganization involving sensorimotor circuits has been described [32]. Similarly, somatoform disorders show widespread abnormalities in sensorimotor connectivity [33]. Even outside pain conditions, such as in Parkinson's disease, reduced SMN connectivity is linked to deficits in sensory integration required for motor control [34]. Collectively, these findings support the notion that SMN hypoconnectivity represents a transdiagnostic neural mechanism, reflecting a shared marker of impaired sensorimotor integration and centralized pain processing.

The SMN plays a central role in discriminating sensory aspects of pain, including location, intensity, and quality, while coordinating motor responses and muscle tone. Clinically, this may explain why patients report more severe sensory pain: The network responsible for processing discriminative pain features is less synchronized with modulatory centers, amplifying the sensory dimension of pain. This interpretation is reinforced by intervention studies targeting the SMN. Flodin [35] demonstrated that supervised exercise improved FM symptoms and partially normalized connectivity between the right anterior insula and the left primary sensorimotor cortex. Likewise, Silva [36] showed that transcranial magnetic stimulation (TMS) applied to the motor cortex significantly reduced pain in FM patients. Clinical improvement following TMS was associated with altered connectivity in sensory and affective networks, including modified synchrony between the ventromedial prefrontal cortex, M1, the insula, and other pain‐related regions. Supporting this, Argaman et al. [37] demonstrated that neuromodulation can alter functional connectivity in both sensory and affective circuits, modulating the subjective pain experience. Together, these findings highlight that improving SMN functionality can mitigate pain, emphasizing the clinical importance of sensorimotor dysfunction in FM.

The findings of this study indicate that FM is associated with a specific reduction in functional connectivity within the right SMN, which correlates negatively with the sensory dimension of pain. Although the direction of this association aligns with prior literature, the effect size was modest and reached only the threshold of statistical significance, suggesting a trend‐level relationship that should be interpreted with caution. Importantly, we did not observe significant correlations between SMN connectivity and the affective, evaluative, or miscellaneous dimensions of the MPQ. This selective association points to a potentially specific role of the SMN in modulating the sensory‐discriminative component of pain rather than its emotional or cognitive aspects. This aligns with the anatomical and functional role of the SMN in processing somatosensory input and coordinating motor responses and supports the interpretation that altered integration in this network may amplify the sensory salience of pain in FM. These results suggest that dysfunction in this network contributes to the central amplification mechanisms characteristic of the syndrome, particularly its sensory‐discriminative component. The data reinforce the hypothesis that FM involves neurofunctional changes concentrated in sensory circuits. These findings deepen the understanding of the neural substrates of chronic pain and may support the development of diagnostic and therapeutic strategies targeting functional modulation of these networks.

In chronic pain conditions, including FM, a recurring pattern emerges. The SMN no longer functions solely as a sensory input system; instead, it becomes abnormally coupled with the DMN and the salience network (SN), thereby linking bodily representation, salience processing, and self‐referential thought [38]. In FM, connectivity between sensorimotor regions and the anterior insular cortex (a key SN hub) is elevated and correlates with clinical pain intensity [39, 40], and a similar connectivity pattern occurs in chronic low back pain [38]. FM and other chronic pain disorders also show increased DMN connectivity with the anterior insula (within the SN) and with somatosensory cortices, regions mediating the sensory‐discriminative aspect of pain [41, 42]. This pattern suggests a greater “intrusion” of somatosensory signals into self‐referential processes [38, 40]. In our results, the DMN and SN did not show any significant connectivity changes, likely due to the stringent significance threshold and multiple‐comparison correction applied. Furthermore, FM is a heterogeneous condition, and its network connectivity alterations can vary according to patient characteristics and experimental conditions. For example, recent evidence indicates that differences in DMN connectivity in FM are most pronounced when patients experience pain during the neuroimaging session and may not be detectable when patients are pain‐free [41].

This study presents some limitations that should be considered when interpreting the findings. First, the cross‐sectional design precludes establishing causal relationships between functional connectivity alterations and clinical symptoms. Second, although the sample was matched for age and education, the relatively small sample size may limit the generalizability of the results. Third, the study included only female participants, which restricts the extrapolation of these findings to male patients with FM, given known sex‐related differences in pain processing and brain functional organization. Future directions include the application of integrative, AI‐based multimodal approaches combining functional connectivity, cortical thickness, morphometry, clinical variables, and psychometric measures. Machine‐learning predictive models could help identify neurofunctional profiles associated with clinical subtypes of FM and contribute to the development of more robust diagnostic markers. Such advances may support personalized treatment strategies and more precise monitoring of therapeutic interventions.

## Funding

This study received institutional support from the Federal University of Rio de Janeiro (UFRJ). The authors also acknowledge partial support from Brazilian research agencies, including the Coordenação de Aperfeiçoamento de Pessoal de Nível Superior (CAPES—Brazil), the Conselho Nacional de Desenvolvimento Científico e Tecnológico (CNPq—Brazil), and the Fundação Carlos Chagas Filho de Amparo à Pesquisa do Estado do Rio de Janeiro (FAPERJ).

## Disclosure

The authors declare no commercial or financial relationships that could be construed as a potential conflict of interest. The authors report no equity interests, stock ownership, consulting roles, speaking fees, paid expert testimony, patent applications, or industry‐sponsored research related to the content of this manuscript.

## Conflicts of Interest

The authors declare no conflicts of interest.
